# MAP17 (PDZK1IP1) and pH2AX are potential predictive biomarkers for rectal cancer treatment efficacy

**DOI:** 10.18632/oncotarget.26010

**Published:** 2018-08-31

**Authors:** Maria Rivero, Javier Peinado-Serrano, Sandra Muñoz-Galvan, Asuncion Espinosa-Sánchez, Elisa Suarez-Martinez, Blanca Felipe-Abrio, Maria Carmen Fernández-Fernández, Maria Jose Ortiz, Amancio Carnero

**Affiliations:** ^1^ Instituto de Biomedicina de Sevilla, HUVR, CSIC, Universidad de Sevilla, Seville, Spain; ^2^ Department of Radiation Oncology, HUVR, Seville, Spain; ^3^ CIBER de Cáncer, ISCIII, Madrid, Spain; ^4^ Department of Pathology, HUVR, Seville, Spain

**Keywords:** MAP17, colorectal cancer, biomarkers

## Abstract

Rectal cancer represents approximately 10% of cancers worldwide. Preoperative chemoradiotherapy increases complete pathologic response and local control, although it offers a poor advantage in survivorship and sphincter saving compared with that of radiotherapy alone. After preoperative chemoradiotherapy, approximately 20% of patients with rectal cancer achieve a pathologic complete response to the removed surgical specimen; this response may be related to a better prognosis and an improvement in disease-free survival. However, better biomarkers to predict response and new targets are needed to stratify patients and obtain better response rates.

MAP17 (PDZK1IP1) is a small, 17 kDa non-glycosylated membrane protein located in the plasma membrane and Golgi apparatus and is overexpressed in a wide variety of human carcinomas. MAP17 has been proposed as a predictive biomarker for reactive oxygen species, ROS, inducing treatments in cervical tumors or laryngeal carcinoma. Due to the increase in ROS, MAP17 is also associated with the marker of DNA damage, phosphoH2AX (pH2AX). In the present manuscript, we examined the values of MAP17 and pH2AX as surrogate biomarkers of the response in rectal tumors. MAP17 expression after preoperative chemoradiotherapy is able to predict the response to chemoradiotherapy, similar to the increase in pH2AX. Furthermore, we explored whether we can identify molecular targeted therapies that could help improve the response of these tumors to radiotherapy. In this sense, we found that the inhibition of DNA damage with olaparib increased the response to radio- and chemotherapy, specifically in tumors with high levels of pH2AX and MAP17.

## INTRODUCTION

Rectal cancer represents approximately 10% of cancers worldwide [1]. In 2012, there were 446.801 newly diagnosed cases and 214.727 deaths attributed to this entity in Europe [1]. Colorectal cancer was estimated to be the most frequent neoplasm diagnosed in Spain in 2015 [2]. The standard of care in patients with locally advanced rectal cancer is preoperative chemoradiotherapy before surgical excision. The addition of preoperative radiation therapy improves local control and survival in this set of patients [3–8]. Preoperative chemoradiotherapy increases the complete pathologic response and local control, although it offers a poor advantage in survivorship and sphincter saving compared with that of radiotherapy alone [9–13]. After preoperative chemoradiotherapy, approximately 20% of patients with rectal cancer achieve a pathologic complete response in the surgical specimen removed, which may be related with a better prognosis and an improvement in disease-free survival [14]. However, better biomarkers to predict response and new targets are needed to stratify patients and to obtain better response rates.

MAP17 is a small, 17 kDa non-glycosylated membrane protein located in the plasma membrane and Golgi apparatus [15]. Although its physiological role is not fully clear, different studies have shown that it stimulates sodium-glucose linked transporter (SGLT), thereby increasing glucose and mannose uptake in Xenopus ovocites [15] and human tumor cells [16].

MAP17 is overexpressed in a wide variety of human carcinomas [16]. According to a study by Guijarro *et al*. [16] on carcinoma tumor samples from different localizations, MAP17 overexpression is >70% in the ovary, colon, stomach, cervix and thyroid gland and approximately 50% in the lung, uterus and rectum. Furthermore, overexpression of the protein strongly correlates with tumor progression in colon, prostate, sarcomas and ovarian carcinomas (*P* < 0.0001) [16].

Tumor cells that overexpress MAP17 show phenotypic advantages with enhanced proliferative capabilities, decreased apoptotic sensitivity and increased migration [17]. The mechanism responsible for the increased tumor capabilities of cells expressing MAP17 has not yet been described. MAP17 overexpression *in vitro* activates the Notch pathway in tumor cells, leading to an increase in the stem cell pool [18]. This aberrant signaling activation may be present in a large percentage of tumors [18]. A correlation between MAP17 expression and an inflammatory phenotype in tumors and other inflammatory diseases has also been described. Immunohistochemical analysis has confirmed local inflammation, even at the site of MAP17 expression in tumors [19]. Chronic inflammation is also a cause of neoplastic transformation and progression; therefore, it is likely that MAP17 plays an important role in cancer development by regulating the immune microenvironment [19].

This increased malignant behavior is associated with an increase in reactive oxygen species (ROS) production, and treatment of cells with antioxidants reduces their tumorigenic properties [17]. ROS play a fundamental role in cellular physiology. They promote both cell proliferation and growth and cell death, which is a highly efficacious tool in cancer treatment. This dual mechanism has been related with ROS concentrations in the cellular environment. At low levels, they are involved in maintaining cellular homeostasis and regulate cellular physiological processes such as proliferation and apoptosis [20]. When the concentration of ROS increases, they act as oncogene activators [21] and as intracellular second messengers for proliferation and cell growth [22, 23]. However, further increases in ROS (close to threshold levels) may induce a toxic environment and turn the physiology of cells towards apoptosis [24, 25].

The ectopic expression of MAP17 increases glucose and mannose uptake, generating an increase in ROS levels as a product of increased metabolism [17]. A direct link between MAP17 and the terminal domain of glucose transporters is also possible, altering ion exchanges and the intracellular redox-balance [26]. Tumors expressing high levels of MAP17 may benefit from therapies that increase oxidative stress. These tumors show increased ROS production and could cross the threshold toxic level easier than non-tumor cells with oxidative treatments [26], which has been observed in tumor types subjected to ROS-inducing treatments.

MAP17 expression was detected in approximately 70% of tumors from more than 200 cervical tumor samples obtained from biopsies prior to treatment. After treatment with cisplatin plus radiotherapy, high levels of MAP17 were related with improved patient survival [27]. Therefore, high levels of MAP17 could serve as a marker for good prognosis in patients with cervical tumors after cisplatin plus radiotherapy treatment [27].

Similarly, MAP17 has also been proposed as a predictive biomarker for laryngeal carcinoma. Patients with larynx cancer and high MAP17 expression in pretreatment biopsies showed better outcomes than those with low MAP17 expression [28]. MAP17 expression was associated with overall survival (OS) (*p* < 0.001), laryngoesophageal dysfunction-free survival (*p* = 0.002) and locoregional control (*p* = 0.016) [28]. The same study found a positive correlation between MAP17 expression and SGLT (*p* = 0.022) and high levels of MAP17/SGLT in combination with an increase in OS (*p* = 0,028) [28]. MAP17 is also associated with the marker of DNA damage, phosphoH2AX (pH2AX). When pH2AX was evaluated in combination with MAP17, higher expression of both potential biomarkers was associated with an improvement in laryngoesophageal dysfunction-free survival (61.35 vs. 32.2 months, *p* = 0.05) and OS (66.6 vs 39.8 months, *p* = 0.01) [29]. In tumors with MAP17 overexpression, pH2AX, which acts as a surrogate of tumor damage, could be induced by an increase in ROS. [29] Histone H2AX is a variant of histone H2A and is implicated in DNA repair. H2AX becomes phosphorylated in response to DNA double-strand breaks and is then called gamma-H2AX (γH2AX or pH2AX), which participates in the recruitment of DNA repair proteins; it can be phosphorylated by kinases of the PI3-family, such as ataxia telangiectasia mutated (ATM) and ATMRad3- related (ATR), in response to DNA damage. pH2AX has been employed as an indicator of unrepaired DNA damage and has been proposed as a sensitive marker of cancer progression and response to cancer therapies [30].

In the present study, we investigated whether MAP17 and pH2AX expression levels can be detected in locally advanced rectal cancer after preoperative chemoradiotherapy and their relationship with disease-free survival (DFS) and OS prognostic values. Furthermore, we explored whether we can identify molecular targeted therapies that could help improve the response of these tumors to radiotherapy.

## RESULTS

### MAP17 expression in rectal tumor samples

**Figure 1:**
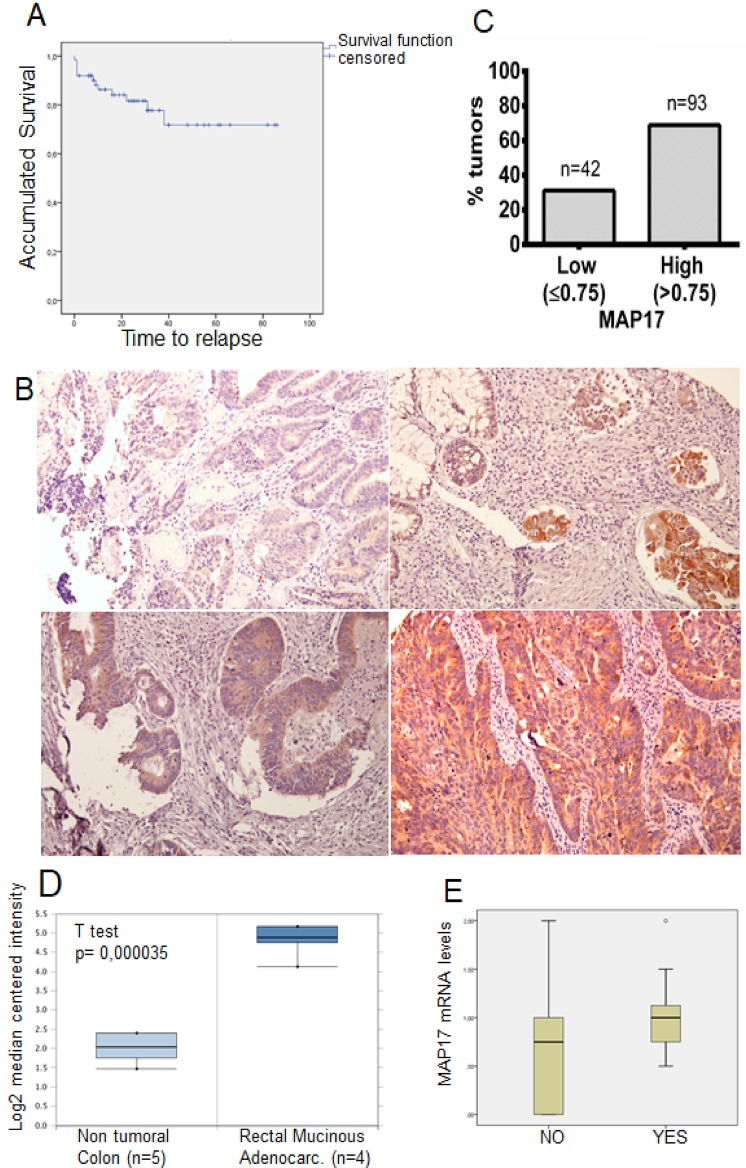
MAP17 upregulation in rectal tumors after concurrent chemoradiotherapy (**A**) Overall survival analysis of the rectal tumor cohort (Supplementary Table 1) used in this study. (**B**) Representative pictures of MAP17-stained rectal tumor samples. An ROC curve was used to identify the cut-off point (0.75). (**C**) Percentage of tumors positive and negative for MAP17 in our cohort of rectal tumor samples (*n* = 135). (**D**) MAP17 mRNA expression levels in non-tumor colon (*n* = 5) and rectal mucinous adenocarcinomas (*n* = 4). (**E**) MAP17 mRNA expression levels in samples from patients without metastasis (NO) vs patients with metastasis (YES).

Our cohort of 135 samples from patients with locally advanced rectal cancer who received preoperative chemoradiotherapy in the same institution from 2005 to 2014 (Supplementary Table 1) was evaluated for OS for comparison with published data. In our cohort, we found a 5-year OS of 75% (Figure 1A), which is similar to other published cohorts [31, 32].

Positive MAP17 expression was evaluated by immunohistochemistry (Figure 1B). Increased MAP17 protein expression in the tumor samples was defined to occur when the percentage of stained tumor was higher than 0.75 (0–3, as indicated in the M&M) compared to that of non-tumor samples [27, 28], and low expression was defined by a value lower or equal to 0.75. Under these conditions, 93 (69%) tumor samples exhibited high expression of MAP17 and 42 (31%) exhibited low expression (Figure 1C). Similar data were obtained when the mRNA was analyzed in publicly accessible databases, such as the Kaiser colon [33] through the Oncomine analysis portal (Figure 1D). The analysis of our cohort also revealed that the levels of MAP17 were associated with the development of metastases (*p* = 0.07). Although it does not reach statistical significance, shows a clear trend (Figure 1E).

### MAP17 expression and prognostic values

**Figure 2:**
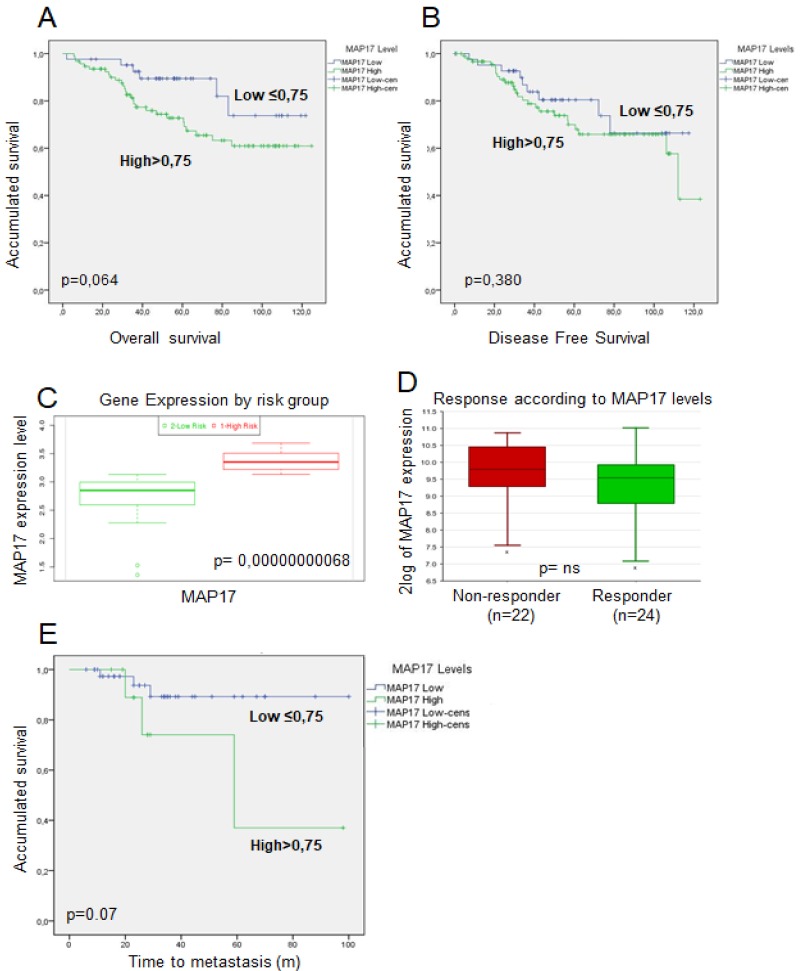
Prognosis analysis of MAP17 expression in rectal tumors after concurrent chemoradiotherapy (**A**, **B**) Association between MAP17 protein expression assessed by IHC to survival in the rectal tumor cohort. (A) Overall survival (OS) and (B) disease-free survival (DFS) Kaplan-Meier survival curves based on MAP17 protein expression. (**C**) mRNA MAP17 expression by risk group in TCGA rectal tumors cohort. (**D**) mRNA MAP17 expression by responders or non-responders in TCGA rectal tumors cohort. (**E**) Kaplan–Meier accumulated survival curve according to time to metastasis by MAP17 mRNA expression.

Patients with low MAP17 expression also showed a better OS (*p* = 0.064) and DFS (*p* = 0.380) compared with those with high expression (Figure 2A and 2B). Although these data do not reach statistical significance, they show a clear trend, individuals with higher levels of MAP17 are at a higher risk and show a worse response to anticancer treatments (Figure 2C and 2D) than those with lower levels of MAP17.

Since MAP17 levels were associated with metastases, we analyzed whether MAP17 is linked to this phenotype or whether its diagnostic capability is independent of the metastases and analyzed its survival probability. Patients with metastases and high MAP17 levels showed worse survival probability than those with low MAP17 levels (*p* = 0.7) (Figure 2E) Although it does not reach statistical significance, shows a clear trend. Therefore, MAP17 is an independent prognostic factor of metastases.

### Pathways enriched in genes correlating with MAP17 expression in rectal tumors

**Figure 3:**
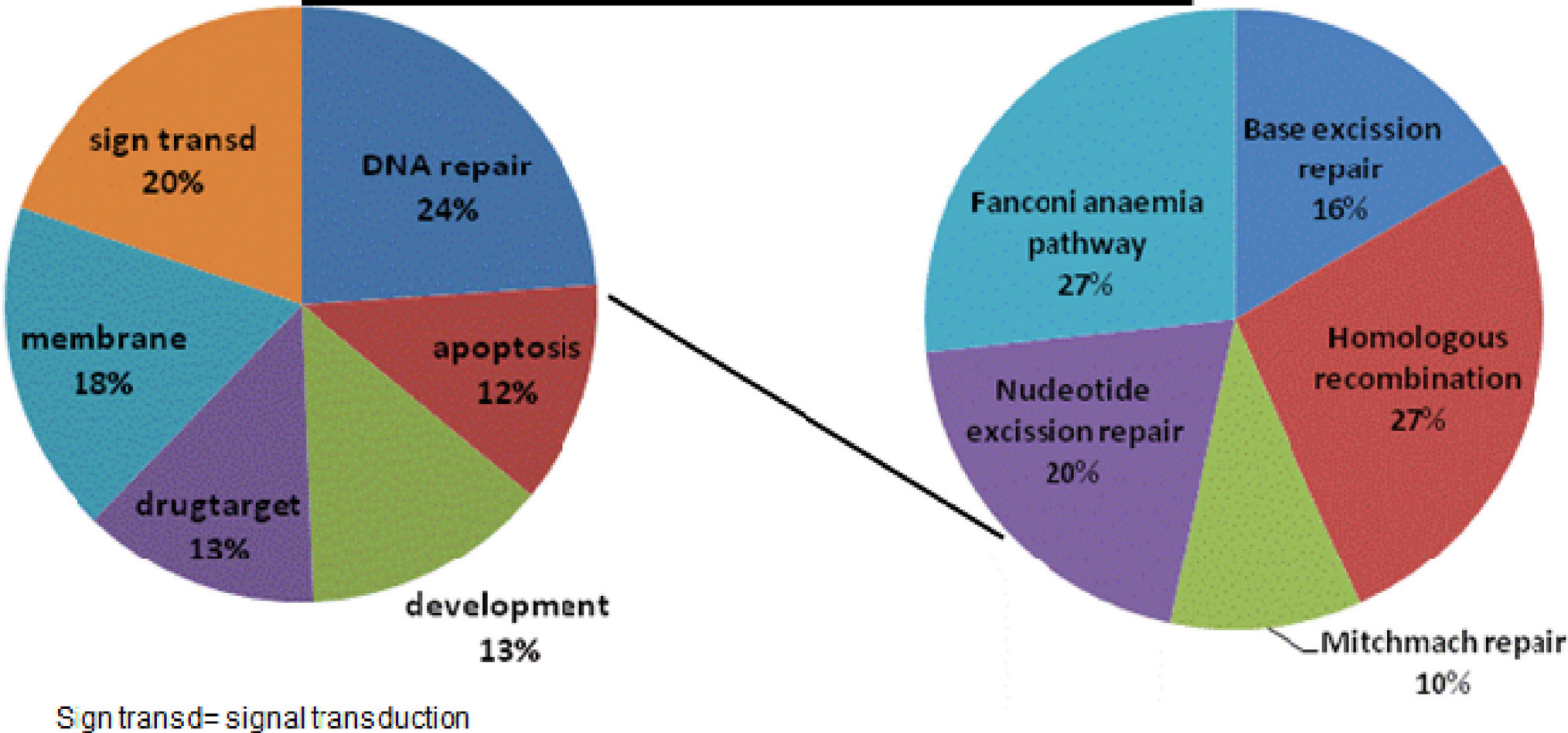
Gene Ontology pathways correlating with MAP17 expression Genes that correlated with MAP17 expression (Pearson *r* < −0.35, *r* > 0.35) were selected. Gene Ontology analysis was applied, and the most representative signaling pathways are presented.

Next, we used the R2 bioinformatics platform to analyze genes and pathways that may be associated with MAP17 expression in rectal tumors. Altered genes associated with MAP17 expression were mainly related with DNA damage and DNA repair functions (Figure 3). These genes were also related with signal transduction, membrane proteins, drug targets and development. Among the genes related to DNA damage and DNA repair functions, we found genes involved in the Fanconi anemia pathway (27%) homologous recombination (27%), nucleotide excision repair (20%), base excision repair (16%) and mismatch repair (10%).

### Phosphorylation of H2AX (pH2AX) in rectal tumor samples

**Figure 4:**
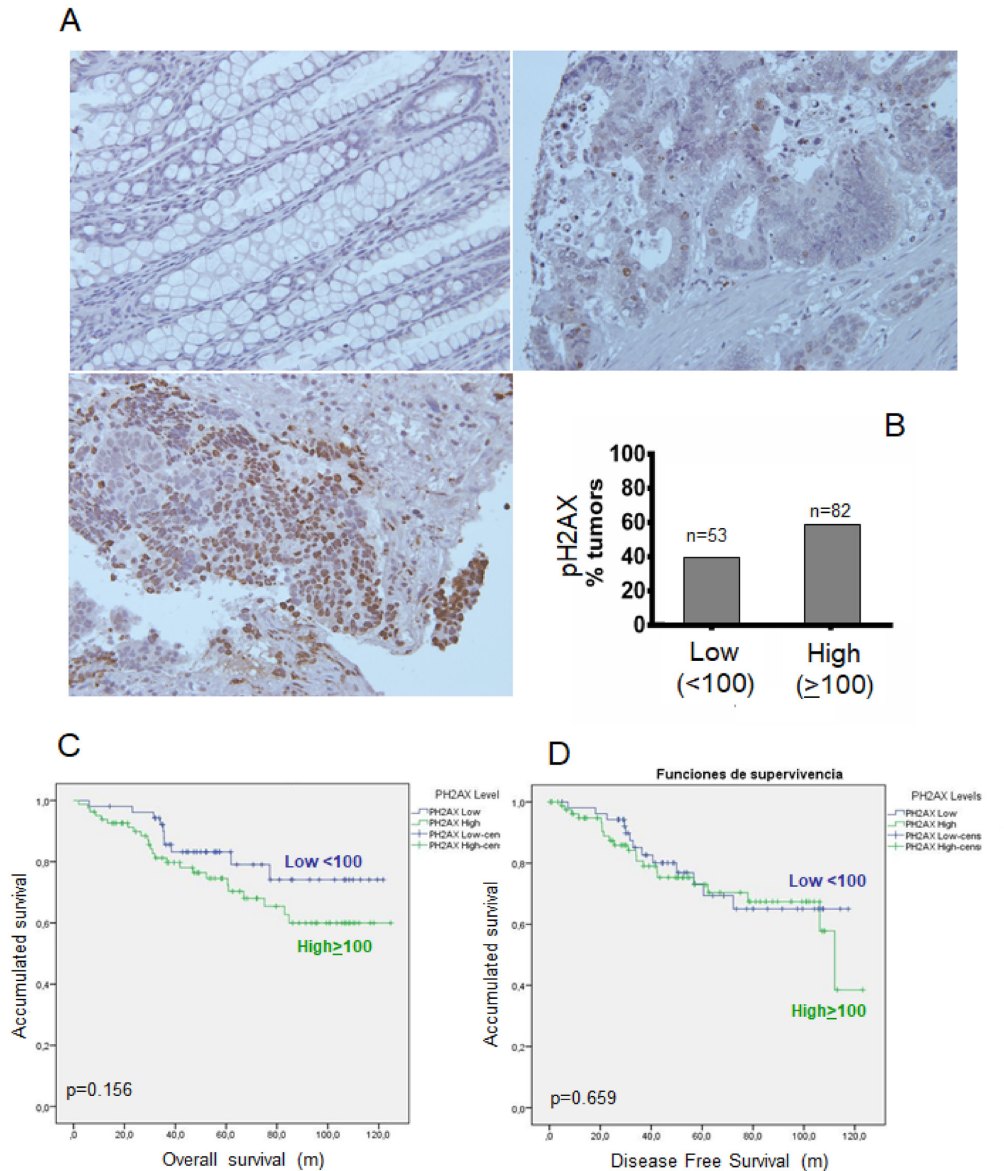
Phosphorylation of γH2AX (p-H2AX) in rectal tumors after concurrent chemoradiotherapy (**A**) Representative pictures of MAP17-stained rectal tumor samples. (**B**) Percentage of tumors positive and negative for MAP17 in our cohort of rectal tumor samples (*n* = 135). An ROC curve was used to identify the cut-off point (100). (**C**) Overall survival (left) and disease-free survival (right) analysis of the rectal tumor cohort (Supplementary Table 1) used in this study. (**D**) Disease-free survival analysis of the rectal tumor cohort (Supplementary Table 1) used in this study

Positive pH2AX expression was immunohistochemically examined by double-blind independent observations (Figure 4A). We detected increased pH2AX protein expression in some tumor samples compared to that in non-tumor samples (Figure 4A). The levels of pH2AX expression were scored by multiplying the strength of nuclear staining (0–3) and the percentage of tumor cells with positive nuclei. The cut-off point was considered 100 (<100 as negative, >100 as positive) [29]. Under these conditions, 82 (60%) tumor samples exhibited high expression of pH2AX and 53 (40%) exhibited low expression (Figure 4B).

Low expression of pH2AX was associated with a better prognosis in terms of OS (*p* = 0.156), although this result was not statistically significant (Figure 4C). However, low pH2AX expression was not associated with DFS (*p* = 0.659) (Figure 4D).

**Figure 5:**
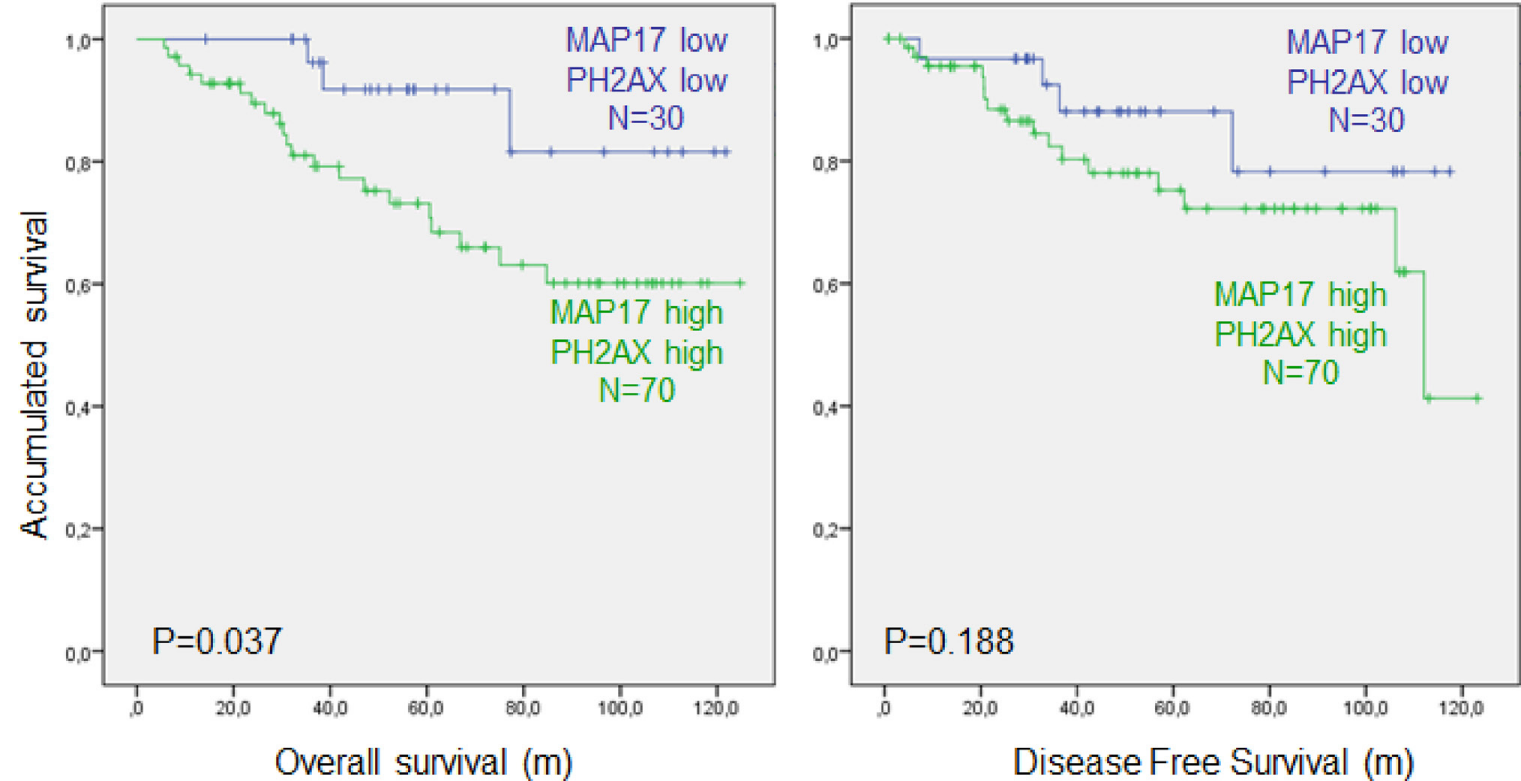
Combined MAP17 and phosphorylation of γH2AX (p-H2AX) analysis of survival in rectal tumors after concurrent chemoradiotherapy Overall survival (left) and disease-free survival (right) analysis of the rectal tumor cohort (Supplementary Table 1) used in this study. Cut-off points are the same as those listed above; high MAP17 (0 > 0.75), high pH2AX (> 100).

When combining pH2AX and MAP17 expression, patients with low marker values of both proteins (pH2AX expression < 100 and MAP17 expression ≤ 0.75) obtained better DFS (*p* = 0.188) and OS (*p* = 0.037) than those with high values (pH2AX ≥100 and MAP17 >0.75) (Figure 5).

### The DNA damage inhibitor olaparib re-sensitizes MAP17- and pH2AX-positive rectal tumors to radiotherapy

It is clear that patients with tumors exhibiting high levels of both MAP17 and pH2AX have a worse prognosis. These results may be related to an increase in the DNA damage response associated with MAP17. It is possible to increase the sensitivity to radiotherapy in these MAP17+pH2AX tumors by inhibiting the response to DNA damage (DDR). This point has been shown in other contexts [34–36] but not in the context of radiotherapy-treated operable locally advanced rectal cancer.

**Figure 6:**
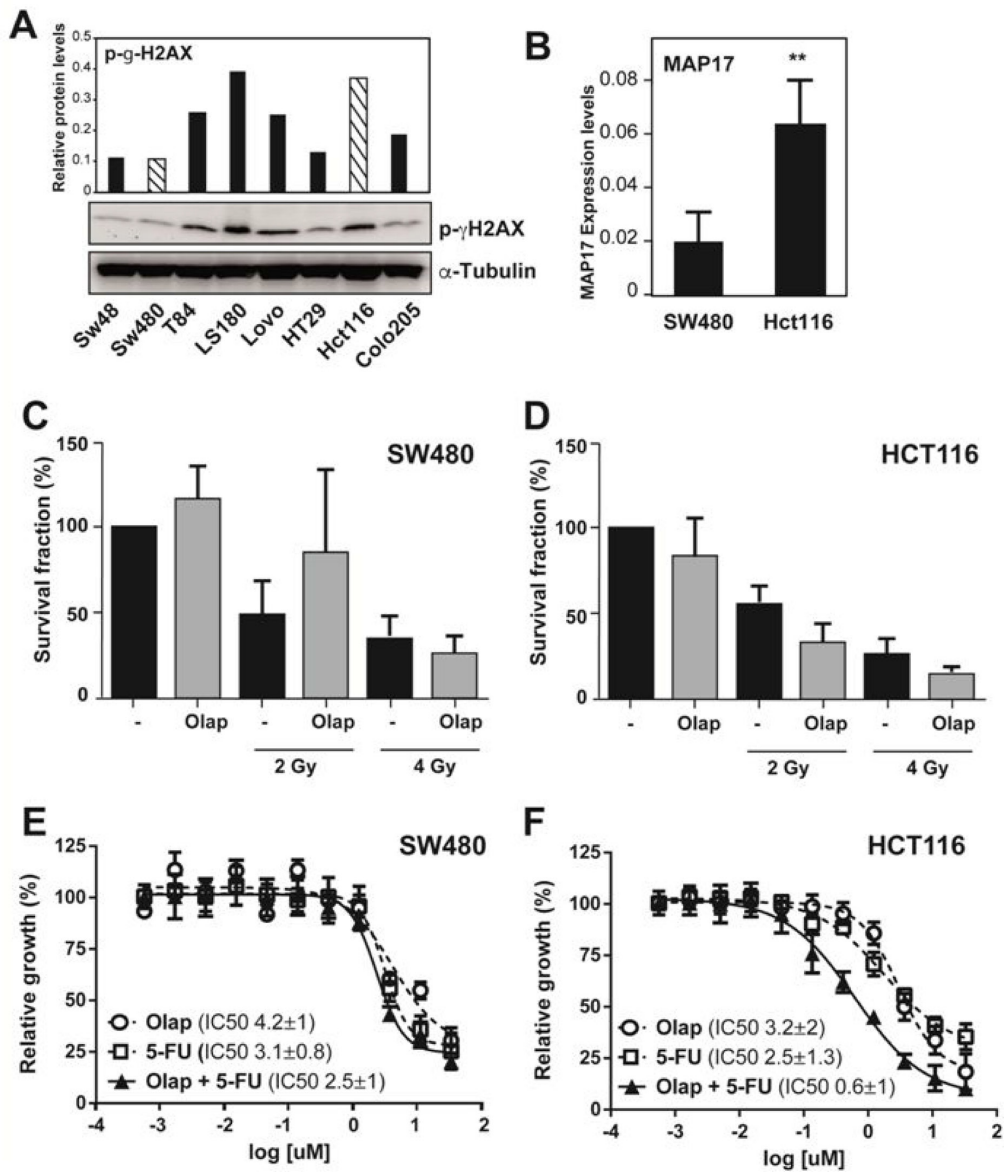
The DNA damage inhibitor olaparib re-sensitizes MAP17- and pH2AX-positive rectal tumors to radiotherapy treatment (**A**) Western blot analysis and relative quantification of the protein levels of phospho-γ-H2AX in colon tumor cell lines. (**B**) RT-qPCR showing the RNA levels of *MAP17* in HCT116 and SW480 colon tumor cell lines. Samples were normalized to *GAPDH*. (**C** and **D**) Percentage of survival fraction after olaparid treatment (10 μM), radiology treatment (2 and 4 Gy) or their combination in HCT116 and SW480 colon tumor cell lines. (**E** and **F**) Determination of the IC_50_ value (concentration of the drug necessary to induce 50% cell death) for SW480 and HCT116 and colon tumor cells lines following treatment with olaparid and/or 5FU.

To prove this point, we explored the levels of MAP17 and pH2AX in a panel of colorectal cell lines (Figure 6A, 6B, Supplementary Table 1). We selected 2 colorectal cell lines, HCT116 with high MAP17 and high pH2AX expression, and SW480, with low MAP17 and pH2AX expression (Figure 6A, 6B). These lines were treated with different doses of radiotherapy either in the presence or absence of the PARP inhibitor olaparib (Figure 6C, 6D). The suboptimal dose selected (10 μM) does not induce any significant decrease in survival. Radiation of 2 Gy alone induced a clear decrease in survival in the HCT116 cell line, which was more pronounced in the presence of olaparib (Figure 6C, 6D). At 4 Gy of radiotherapy treatment, we observed a further decrease in survival that was more pronounced in the presence of olaparib. These data indicate that treatment with suboptimal doses of olaparib prior to radiation treatment may decrease the survival of MAP17+pH2AX-positive tumors (Figure 6C). However, these results are not maintained in SW480, a cell line with low levels of these markers. In this case, olaparib does not induce a further decrease in survival induced by radiotherapy alone (Figure 6F).

Since radiotherapy treatment of rectal tumors is concurrent with chemotherapy, which usually involves 5FU, we determined whether the inhibition of DNA repair by olaparib improves chemotherapy treatment in rectal tumors when applied as concurrent chemotherapy. Both cell lines were treated with different doses of chemotherapy either in the presence or absence of suboptimal doses of olaparib (10 μM) (Figure 6E, 6F). Olaparib treatment did not sensitize SW480 cells, the line with low MAP17 and low pH2AX expression, to 5FU treatment (Figure 6E). However, in the HCT116 cell line with high levels of MAP17 and pH2AX, treatment with suboptimal doses of olaparib caused a significant reduction in the IC_50_ value of 5FU (Figure 6F).

## DISCUSSION

The use of long-course chemoradiation (CRT) or concurrent short-course radiotherapy (SCRT) consistently improves local control rates compared to those from surgery alone, postoperative CRT or postoperative radiotherapy [37, 38]. However, its use does not consistently translate to OS [39].

Therefore, multidisciplinary information has become important when making decisions regarding CRT. These decisions are based on the combination of magnetic resonance imaging (MRI), tumor and patient characteristics and laboratory experimental findings.

Very few robust molecular studies have elucidated the mechanism of chemoradiation resistance. Very few promising predictive biomarkers have been suggested, including EGFR, thymidylate synthase, PI3K, methylation or proteins involved in the ras/MAPK pathway [40–44]. Furthermore, it has been reported that different genomic signatures identify responders and non-responders from whole-genome analysis [39, 42]. However, none of the signatures from the gene microarray profile on locally advanced rectal cancer have been successfully validated as a diagnostic or prognostic tool applicable to routine clinical practice [39].

Our work shows that patients with high MAP17 are associated with worse OS and the presence of metastasis. Furthermore, the associated high levels of MAP17 and pH2AX showed better prognostic capability than MAP17 or pH2AX alone. The fact that the combination of MAP17 and pH2AX has better prognostic capability in rectal tumors is consistent with others in laryngeal cancer in which high levels of MAP17 (and to a greater extent, high MAP17 and high pH2AX) correlated with improved patient survival after treatment [28, 45, 46].

In this setting, our analysis in rectal cancer showed a clear but not statistically significant relationship between low MAP17 protein expression and increased OS, suggesting that MAP17 expression is an independent biomarker for survival. In fact, high MAP17 levels demonstrated worse OS than low levels.

pH2AX was also related with OS in the same cohort of 135 patients with rectal cancer. Our data suggest that inherent DDR pathway activation (measured by the endpoint of γH2AX phosphorylation) is a valuable prognostic marker in patients with rectal carcinoma who received radiation therapy.

It has been proposed that the DDR network may serve as an inducible barrier to control the initial steps of tumor development by inducing p53-dependent senescence or apoptosis [46–49]. Ongoing chronic DDR activation favors the outgrowth of malignant clones with genetic or epigenetic defects in a DNA repair mechanism, such as those involved in the DDR pathway [49]. Our samples from already malignant tumors (stages II-IV) correlated with a worse onset of disease. It is likely that DNA damage defects that induce DDR activation are carried through the malignant process and it is possible that other proteins are mutated in the process, resulting in the DNA-damaging effect of radiotherapy.

We also showed that the combination of MAP17 and pH2AX has a predictive role in patients with rectal cancer treated with CRT. Taking into account the combined analysis of pH2AX and MAP17, we hypothesize that the DDR pathway plays an essential role in the predictive outcome of rectal cancer. Furthermore, proof of concept analysis of tumor cells treated with olaparib indicated that DNA repair pathway inhibition sensitizes tumor cells with high MAP17 and pH2AX levels to radiotherapy and concurrent chemotherapy.

High MAP17 levels are associated with OS. MAP17 increases endogenous ROS [26, 27, 50], which induce DNA damage [51]. Data from our study and others suggest that high levels of MAP17 induce ROS, which increase DNA damage and DDR signaling; this is denoted by higher pH2AX levels [45, 51]. Upon the inhibition of DNA damage or a further increase in damage by RT treatment, tumors are more likely to undergo apoptosis. Furthermore, proof of principle experiments *in vitro* demonstrated that DNA damage inhibitors such as olaparib increased the sensitivity of MAP17-expressing cells, confirming the relevance of the oxidative status of the tumors in response to radiation. Patients who are unlikely to respond could be offered an alternative approach to therapy by co-adjuvant or concurrent chemotherapy combined with inhibition of the DNA damage response pathway.

Therefore, our data confirm that pH2AX (and its combination with MAP17) levels, is a marker of structural DNA damage in rectal tumors and thus may serve as a novel valuable and easily applicable predictive biomarker for rectal carcinoma.

In conclusion, current treatment depends on clinical and laboratory predictive and prognostic markers. Our study focused on the use of two predictive markers to explain some variability in response to standard treatment. MAP17 and pH2AX, either alone or in combination, levels obtained after RT and co-adjuvant therapy or concurrent chemotherapy treatment are predictive markers to predict the responses of rectal tumors. Furthermore, our work offers an adjusted therapy to patients with a combination of these biomarkers (high MAP17 + high pH2AX) by the addition of olaparib (or other DNA repair inhibitors + RT treatment). In future trials, assessing CRT and neo-, coadjuvant or concurrent chemotherapy for rectal cancer should be evaluated prospectively. It is clear that there is a biological basis to support the use of olaparib as a co-adjuvant or concurrent chemotherapy in patients with tumors with high MAP17+pH2AX and treated with radiation + 5FU, while patients with markers predicting a poor response could be offered an adjusted therapy in terms of agents or treatment sequence.

## MATERIALS AND METHODS

### Study approval

All patients provided written informed consent according to the protocol approved by the local ethics committee (CEI 0309-N-15). All tissue samples and patient information were treated according to the Declaration of Helsinki.

### Cohort description and treatment

We evaluated tumor samples from 135 patients with locally advanced rectal cancer who received preoperative chemoradiotherapy in the same institution from 2005 to 2014 (Supplementary Table 1).

Eligibility criteria included patients with locally advanced rectal cancer T3-4 N+ M0 (stages II-III) who completed the neoadjuvant chemoradiotherapy plan before resection with curative intention. We excluded patients with synchronous metastases at diagnosis or with metastatic disease before treatment. The patients included 43 women and 92 men. The median diagnostic age was 68 years old. Fifty-four tumors were localized in the low rectum (3–5 cm from the anal verge), 45 were localized in the medium rectum (6–9 cm) and 34 were localized in the high rectum (10–15 cm). In two patients, the localization was not recorded in the medical history. Before induction treatment, 28 patients presented with tumors with a histological grade of 1, 67 presented with a grade of 2, 5 presented with a grade of 3 and, in 35 patients, these data were missing. One patient presented with TNM stage I before neoadjuvant treatment and surgery, 23 presented with IIA stage, 3 patients presented with IIB stage, 10 patients presented with IIC stage, 7 patients presented with IIIA stage, 67 patients presented with IIIB stage, 22 patients presented with IIIC stage, and 2 patients presented with stage IVa. All patients received the same neoadjuvant chemoradiotherapy treatment based on three-dimensional conformal radiation therapy (3D-CRT) achieving doses of 45–50.4 Gy with concurrent chemotherapy. Concurrent chemotherapy regimens included continuous infusion of 5-fluorouracil (225 mg/m^2^) over 24 hours 5 or 7 days/week or capecitabine (825 mg/m^2^) twice daily 5 days/week during radiation therapy. Most patients underwent surgery 4–6 weeks after neoadjuvant treatment.

Eighty-nine patients were managed with low anterior resection, 41 with abdominoperineal resection, and 5 with another procedure. After wound healing and overall recovery, most patients received adjuvant chemotherapy with XELOX (capecitabine plus oxaliplatin) or capecitabine monotherapy.

After a median follow-up of 45 months, 34 patients experienced tumor recurrence: 11 presented with local relapse, 7 presented with hepatic disease, 7 presented with lung disease, 2 presented with local relapse and synchronous hepatic metastases, 3 presented with local relapse and synchronous lung metastases, 1 presented with local relapse and peritoneal disease, and 3 presented with distant metastases in a different location. In the same period, 34 patients passed away. The mean OS was 91.99 months (95% confidence interval [CI] 83.78–100.20) and the mean DFS was 81.96 months (95% CI 73.17–90.76).

### Tissue acquirement and preparation

Formalin-fixed, paraffin-embedded tissue sections from 135 rectal tumors were selected. Histological characterization of all samples was assessed by hematoxylin and eosin staining. We constructed tissue microarrays (TMAs) with two representative samples of each tumor and 3 control samples of non-tumor rectal tissues.

These tissue samples were solicited to the Andalusian Public Health System Biobank under the current normative, and all samples were owned by patients who expressed their agreement in writing for the donation and handling of the samples for scientific purposes.

### Immunohistochemistry

Three-micrometer slices were sectioned from the TMA block and applied to coated immunochemistry slides (DAKO, Glostrup, Denmark). The slides were baked overnight in a 56°C oven, deparaffinized in xylene for 20 min, rehydrated through a graded ethanol series and washed with PBS. A heat-induced epitope retrieval step was performed by heating the slide in a solution of sodium citrate buffer (pH 6.5) for 2 min in a conventional pressure cooker. After heating, the slides were incubated with proteinase K for 10 min and rinsed in cool running water for 5 min. Endogenous peroxide activity was quenched with 1.5% hydrogen peroxide (DAKO) in methanol for 10 min. Incubation with the primary antibodies, anti-MAP17 (1:4) [29–33] and anti-phospho-g-H2AX (phospho S139)(ab11174 from Abcam), was performed for 40 min. After incubation, immunodetection was performed with the EnVision (DAKO) visualization system using diaminobenzidine chromogen as the substrate according to the manufacturer's instructions. Immunostaining was performed in a TechMate 500 automatic immunostaining device (DAKO) and measured through a double-blind visual assessment using microscopic observation according to the anatomopathological experience of the pathologists. Sample scoring was performed by semiquantitative microscopic analysis while considering the number of stained cells and signal intensity. Staining of both MAP17 and phospho-g-H2AX was performed. In both cases, the score obtained by the intensity levels (0, 1, 2 or 3) in positive cells was used. For MAP17, the threshold used was the score of the total cohort obtained by the ROC curve as the most relevant to establish as a dichotomous variable (0.75). For phospho-g-H2AX, there was no staining in any of the non-tumor surrounding tissue, and t positivity was considered when a positive nucleus was observed in the tumor. Immunostaining was evaluated through a double-blind visual assessment using microscopic observation according to the anatomopathological experience of the pathologists.

### Cell lines

Characteristics of the cell lines used are shown in Supplementary Table 2. All cell lines were authenticated and regularly tested for mycoplasma.

### Validation of the response to different doses of radiotherapy

We used the clonogenic assay, which enables the assessment of differences in reproductive viability (capacity of cells to produce progeny, i.e., a single cell to form a colony of 50 or more cells) between control untreated cells and cells that have undergone exposure to ionizing radiation treatment. This method uses the Linear Quadratic model to predict the response of a tumor and normal tissues in the clinic. When the confluence reached approximately 90%, cells were irradiated with 2, 4, 6, or 8 Gy using a 137 Cesium source. After irradiation (24–72 h), the number of cells in each sample was counted carefully using a hemocytometer and diluted such that an appropriate cell number (500–1000) was seeded onto 10-cm dishes. The cells were then incubated from 1 to 4 weeks depending on the cell line and fixed. ImageJ (Fiji Version 1.44a) was used to count colonies. Efficiency and survival fraction were calculated as follows: (number of colonies counted/number of cells plated)/plating efficiency. SF was expressed as SF at 2 Gy if available for comparison to human doses.

### Cytotoxicity studies

Cytotoxicity studies were performed as previously described. Briefly, 3,000 cells/well were seeded onto 96-well plates and allowed to attach and grow for 24 h before treatment. The drugs were diluted with DMSO or water. From here, a “mother plate” was prepared at 200X the final concentration in culture for serial dilutions. Serial dilutions of each drug from an initial 30 μM dose were assayed in a minimum of three independent experiments. The medium was removed from the cells and replaced with 0.2 ml of medium containing the drug to be tested. Each concentration was assayed in triplicate. Two sets of control wells were included on each plate, which consisted of either medium only or medium with the same concentration of DMSO or water. A third control set consisting of untreated cells just before drug addition was also established, which served as the seeding control indicating the number of cells starting the culture. The cells were exposed to the drug for 96 h and subsequently fixed with glutaraldehyde (0.5%) and stained with crystal violet. After dissolving in 20% acetic acid, the resulting absorbance was measured at 595 nM using a microplate reader (Bio-Rad, Hercules, CA, USA), and the cytotoxic effect of each treatment was assessed by calculating the concentration necessary to induce 50% cell death (IC_50_ value) using PRISM software.

### Public databases of clinical samples

To validate our results, we obtained data from publicly available clinical and genomic information from Oncomine (https://powertools.oncomine.com) and TCGA Research Network (https://cancergenome.nih.gov/).

### Statistics

*In vitro* data are presented as the mean ± standard deviation to indicate variation within each group. Statistical analysis was performed with the SPSS statistical package (v19, IBM). The *in vitro* and *in vivo* experiments were analyzed using an unpaired non-parametric Mann–Whitney *U* test or Student´s *t* tests. *P*-values less than 0.05 were considered significant. The Kaplan-Meier method was used for survival analyses of the clinical data and cell line xenograft experiments. A Cox proportional hazards model was used to adjust for explanatory variables and *p*-values were obtained. Type II ANOVA was used to analyze differences in survival among groups. The Cox proportional hazards model was used to obtain the hazard ratio values.

### Variable definitions

DFS was defined as the time from surgery to the time the patient experienced any event (local or distant recurrence and death). OS was defined as the time from surgery to the time the patient died from any cause.

## SUPPLEMENTARY MATERIALS TABLES


