# Supplementary material for: MAP17 (PDZK1IP1) and pH2AX are potential predictive biomarkers for rectal cancer treatment efficacy
Source: Oncotarget. 2018 Aug 31;9(68):32958–71. doi: 10.18632/oncotarget.26010 (PMC6152481; doi:10.18632/oncotarget.26010)
Supplement: Supplementary file 1 [file oncotarget-09-32958-s001.pdf]

MAP17 (PDZK1IP1) and pH2AX are potential predictive biomarkers for rectal cancer treatment efficacy

SUPPLEMENTARY MATERIALS

Supplementary Table 1: Human tumor cohort used in this work

Cohort Description and Treatment	
N	135
Gender	
Women	43
Men	92
Age	Median 68 years old
Tumor localization	
Low rectum (3–5 cm from the anal verge) anal)	54
Medium rectum (6–9 cm)	45
High rectum (10–15 cm)	34
Non recorded in medical history	2
Histology and tumoral grade (pre-treatment biopsy)	
Adenocarcinoma GI	28
Adenocarcinoma GII	67
Adenocarcinoma GIII	5
Non recorded in medical history	35
Pre-treatment stage (TNM)	
I	1
IIA	23
IIB	3
IIC	10
IIIA	7
IIIB	67
IIIC	22
IV	2
Surgery	
Low anterior resection	89
Abdominoperineal resection	41
Other procedures	5
Tumor recurrence	
No events	101
Local relapse	11
Hepatic relapse	7
Lung relapse	7
Local and hepatic relapse	2
Local and lung relapse	3
Local and peritoneal relapse	1
Distant relapse in other localizations	3

Supplementary Table 2: Mutational analysis of the CRC lines used in this study. The mutational status was obtained from COSMID database and confirmed by our own mutational analysis

Cell line	Kras mutation	Braf V600E	PIK3CA mutation
COLO 205	Wild type	Mutated	G914R
HT29	Wild type	Mutated	P449T
HCT116	G13D	Wild Type	H1047R
LS180	G12D	Wild type	H1047R
LOVO	G13D	Wild type	ND
T84	G13D	Wild type	E542K; H1047R
SW480	G12V	Wild type	ND
SW48	Wild type	Wild type	G914R
